# Dose calibration of Gafchromic EBT3 film for Ir-192 brachytherapy source using 3D-printed PLA and ABS plastics

**DOI:** 10.1186/s41205-019-0040-4

**Published:** 2019-02-06

**Authors:** Courtney Oare, Christopher Wilke, Eric Ehler, Damien Mathew, David Sterling, Clara Ferreira

**Affiliations:** University of Minnesota Medical School, 420 Delaware St SE, Minneapolis, MN 55414 USA

**Keywords:** Brachytherapy, Dosimetry, 3D-printing, EBT3 Gafchromic film

## Abstract

3D printing technology has allowed the creation of custom applicators for high dose rate (HDR) brachytherapy, especially for complex anatomy. With conformal therapy comes the need for advanced dosimetric verification. It is important to demonstrate how dose to 3D printed materials can be related to dose to water. This study aimed to determine dose differences and uncertainties using 3D printed PLA and ABS plastics for Radiochromic film calibration in HDR brachytherapy.

Gafchromic EBT3 film pieces were irradiated in water with an Ir-192 source at calculated dose levels ranging from 0 to 800 cGy, to create the control calibration curve. Similarly, film was placed below 3D printed PLA and ABS blocks and irradiated at the same dose levels calculated for water, ranging from 0 to 800 cGy. After a 72-h development time, film pieces were scanned on a flatbed scanner and the median pixel value was recorded in the region of highest dose. This value was converted to net optical density (NOD). A rational function was used to fit a calibration curve in water that relates NOD to dose for red, green, and blue color channels. Based on this fitted curve, ABS and PLA NOD values were used to estimate dose in 3D printed plastics.

From the fitted calibration curve, mean residual error between measured and planned dose to water was less than 1% for each color channel at high dose levels. At high dose levels, ABS and PLA mean residual errors were about 6.9 and 7.8% in the red channel, while 5.2 and 5.7% in the green channel. Combined uncertainties measured to be about 6.9% at high dose levels. This study demonstrated dose differences and uncertainties using 3D printed applicators for HDR Ir-192 brachytherapy.

## Introduction

Custom applicators are useful for treating superficial tumors with high dose rate (HDR) brachytherapy. They allow highly conformal dose delivery, and are especially beneficial for oblique surfaces, such as the face [1, 2]. The Freiburg Flap is a commonly used customizable applicator for treating cutaneous lesions at depths less than 5 mm^3^. While this applicator is useful for treating lesions located on relatively uniform surfaces, it has difficulty conforming to irregularly-shaped structures such as the nose or ear. Recently, the proliferation of 3D printing technology has enabled the creation of customized applicators for the delivery of highly conformal radiotherapy treatment, even in the setting of complex anatomic geometries [1].

With advancement of applicators and conformal therapy comes the need for accurate dosimetry methods. Film dosimetry has proved to be a high-resolution tool for radiotherapy treatment verification. Radiochromic film is useful for HDR brachytherapy quality assurance and is more practical than use of radiographic film or ion chambers [4, 5].

Radiochromic film darkens with radiation exposure and dose, measured by changes in optical density without the need for chemical processing. Radiochromic film dosimetry allows the conversion of net optical density (NOD) to dose based on a source-specific calibration curve in water, or solid water [4]. To create a calibration curve, film is exposed in water at increasing prescribed dose levels. Film response (darkening) is measured in a standard flatbed scanner as NOD. The NOD can be related to dose by a rational function. This fitted function, known as a calibration curve, can then be used as a dosimetry tool for future dose measurements made with Radiochromic film.

Common 3D printed plastics such as acrylic butadiene styrene (ABS) and polymethylmethacrylate (PLA) have been shown to be near-water equivalent [6]. Currently, film calibration for the HDR source is done in water. The process of film calibration in water can be laborious and time consuming. In a busy clinical setting, the use of 3D printed PLA and ABS plastics could offer a faster alternative for obtaining film calibration curves. The goal of this work was to obtain the relationship of doses and uncertainties in 3D printed PLA and ABS plastics when used in lieu of water for film calibration for HDR Iridium-192 brachytherapy sources. Furthermore, as part of a large-scale study to design custom 3D-printed superficial HDR applicators, it is important to demonstrate how dose in 3D printed materials can be related to dose to water.

## Materials and methods

### Source

An HDR remote afterloader (Nucletron, Elekta, Stockholm, Sweden) was used with a MICROSELECTRON V2 Iridium-192 source (Alpha-Omega Services, Inc., Edgerly, LA).

### Film

Gafchromic EBT3 film was used in this study and is the most current model of Radiochromic film available. EBT3 film is near tissue equivalent with an effective atomic number of 7.26 [7]. It has two polyester substrate layers (125 μm) surrounding an active layer (28 μm), creating a symmetric structure that eliminates side orientation dependence. EBT3 film has been shown to be energy independent at doses above 100 cGy, and at energies greater than 100 keV photons [8], making it useful for Ir-192 sources, which have an average photon energy of 380 keV. In addition, EBT3 characteristics such as uniformity, orientation, and energy dependence have been widely studied [9, 10].

All film was obtained from the same lot number, 06131702. Sheets were cut into fourths, creating 10.2 × 12.7 cm^2^ pieces and marked to maintain side and orientation dependence. Film was handled and stored in accordance with American Association of Physicists in Medicine (AAPM) Task Group 55 recommendations and guidelines [11].

### Dose to water measurements

**Fig. 1:**
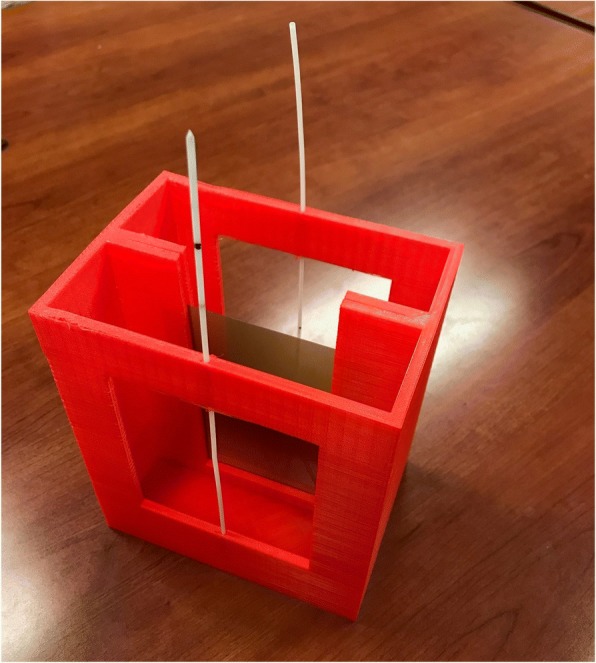
A custom 3D printed film hold was created using ABS. The design can be immersed in a water tank and allows film to be placed between two opposing catheters, 5 cm away from the film’s center

A custom 3D printed holder (made of ABS) was used to take film measurements in water (see Fig. 1). Two opposed catheters were placed 5 cm from the film's center to deliver a total dose of 50, 100, 200, 400, 600, and 800 cGy. Treatment planning software (Oncentra, Elekta, Stockholm, Sweden) calculated doses based on AAPM Task Group 43 (which does not correct for inhomogeneities) [12, 13]. A non-irradiated film sample was used as a 0 cGy background measurement.

### 3D-printed material measurements

**Fig. 2:**
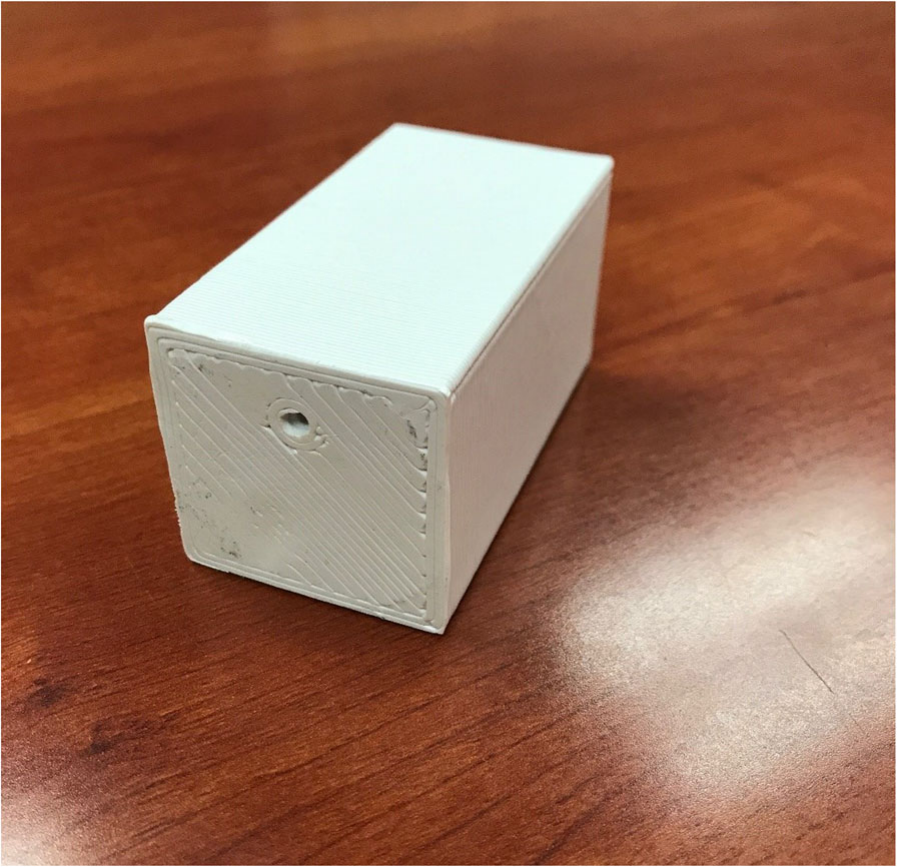
A 3D printed ABS block, 4x4x7 cm^3^, with catheter placement 3 cm from base

**Fig. 3:**
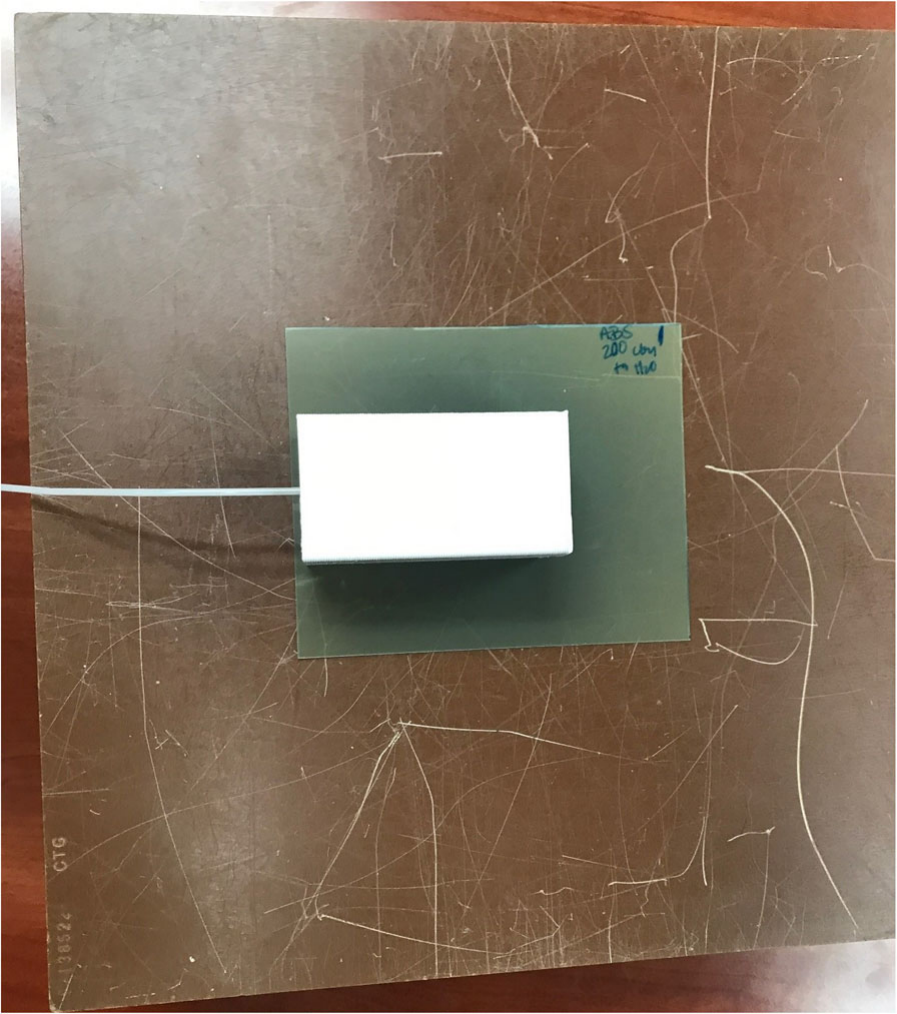
Film is placed 3 cm from the catheter and source, below the 3D printed block

**Fig. 4:**
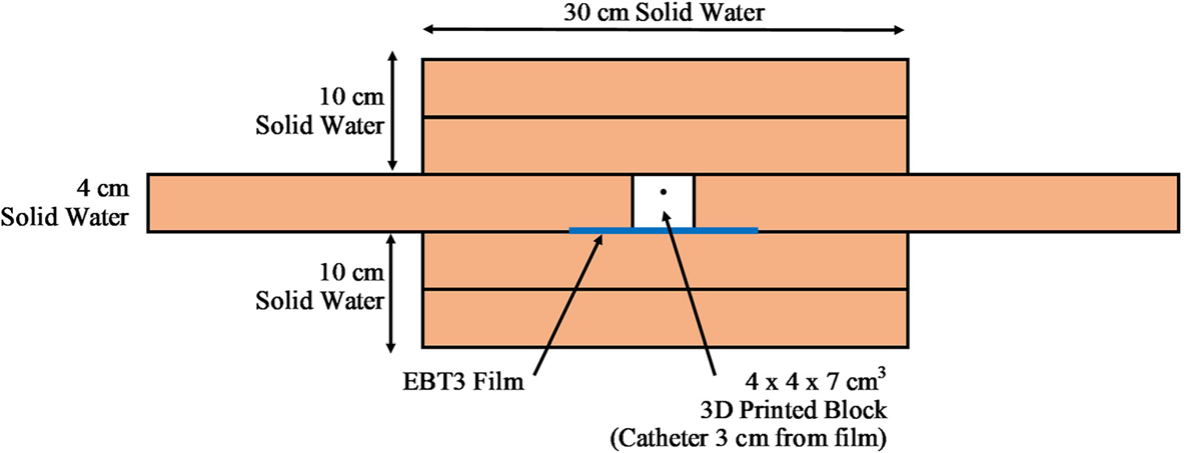
The 3D printed block, and film are surrounded by solid water to create scatter

A 4x4x7 cm^3^ ABS block was 3D printed using a commercially available 3D printer (Taz 6, Aleph Objects Inc., Loveland, CO) with 100% infill and a 0.2 mm layer height. The center of the catheter channel was offset by 1 cm from midline with the film placed along the surface of the block 3 cm from the catheter (see Figs. 2, 3 and 4). The treatment planning software (Oncentra, Elekta, Stockholm, Sweden) was again used to measure dose to water ranging 50–800 cGy to the film. This procedure was repeated for a similar block made of PLA. ABS and PLA were assumed to be near water equivalent in the treatment planning system (TPS) [6].

### Scanning the film

A flatbed scanner (Epson Expression 11000XL, Seiko Epson Corp., Tokyo, Japan) and associated EPSON SCAN, were used to scan the film approximately 72 h post-irradiation. Previous findings have shown minimal variation in NOD beyond a 24 h development time [10]. A foam board positioning template was used to achieve reproducible and uniform position in the center of the scanner. Films were scanned three times each to determine scanning consistency. RBG-positive images were acquired with a spatial resolution of 72 dpi, and a depth of 48 bits (16 bits per color channel). Images were saved in tiff format. The ImageJ software platform (National Institutes of Health, Bethseda, MD) was used to analyze the film with a 9 × 9 pixel region of interest (ROI) selected at the highest point of exposure. The median pixel value (related to image’s intensity) from the ROI was then recorded for each color channel (red/blue/green). Median pixel value can then be converted to NOD by the following formula:

$$ OD=\frac{Pixel\ Value}{2^{16}} $$

$$ NOD={OD}_{Dose}-{OD}_{0\  cGy} $$

### Creating and using a calibration curve

The relationship between film response and dose was determined with a calibration curve based on water measurements. A rational function was used to fit the NOD data with the expected dose from the TPS [14]:

$$ Dose=a+\frac{b}{NOD-c} $$

Using coefficients, a, b, and c to create calibration curve, dose to the can be measured based on changing film response.

### Uncertainty analysis

In this study, uncertainty was determined based on precision of measurements and calculations made. Based on AAPM Task Group 43 uncertainties, both random (type A), and systematic (type B), are measured [12, 13]. Uncertainties were divided into two parts; determining dose from NOD, and film exposure. An estimate of combined uncertainty was calculated using a square root of the sum of squared individual uncertainty components, as recommended by AAPM [13].

## Results

### Calibration curve fit

The parameters shown in Table 1 were fit for red, green, and blue channels, based on the relationship between planned dose and NOD. The dosimetric error was measured as a percent difference between the measured dose with film and the expected dose from the TPS. An absolute mean error was determined to quantify the sensitivity of each channel. Since EBT3 film is less accurate at low doses, the absolute mean error was divided into low dose (≤100 cGy) and high dose (> 100 cGy).

**Table 1:** Fitting Parameters for Calibration Curve

Color Channel	a	b	c
Red	− 582.230	− 436.180	0.757
Green	− 996.825	− 727.023	0.730
Blue	− 1633.021	− 773.091	0.475

A rational function, y = a + (b/(x – c)), was used to fit the data where y is the dose and x is the NOD. Based on these parameters, a calibration curve can be created for each color channel

For water measurements all channels showed high dose mean error less than 1%. At low doses, there were inaccuracies in the calibration curve fit, as shown in Table 2. The blue channel showed the highest error, especially at the 50 cGy dose level. Since the red and green channels showed little dosimetric error at both high (≤0.1%) and low (< 5.0%) dose levels, they will be used for the remainder of the analysis. Previous studies emphasize that red and green channels were most sensitive for high dose levels [4, 10].

**Table 2:** Dose Measured in Water and Residual Error

TPS Dose (cGy)	Red (cGy)	Error (%)	Green (cy)	Error (%)	Blue (cGy)	Error (%)
0	−6.1	–	− 0.6	–	−4.9	–
50	53.5	7.0	52.1	4.2	60.0	19.9
100	102.5	2.5	97.8	−2.2	98.07	−1.8
200	203.0	1.5	199.3	−0.4	197.8	−0.1
400	397.0	−0.8	400.3	0.1	395.0	−1.2
600	596.4	−0.6	602.6	0.4	600.7	0.2
800	801.5	0.2	798.1	−0.2	796.2	−0.4
Low Dose Mean		4.8		3.2		10.9
High Dose Mean		0.8		0.3		0.7

Dose to water was measured based on calibration curve fit (in Table 1). The residual error was determined as the percent difference between the planned and the measured dose to water. An absolute mean error was calculated for each color channel. The mean error was split into low dose (< 100 cGy) and high dose (> 100 cGy) ranges

### Dose measurements in PLA and ABS

**Fig. 5:**
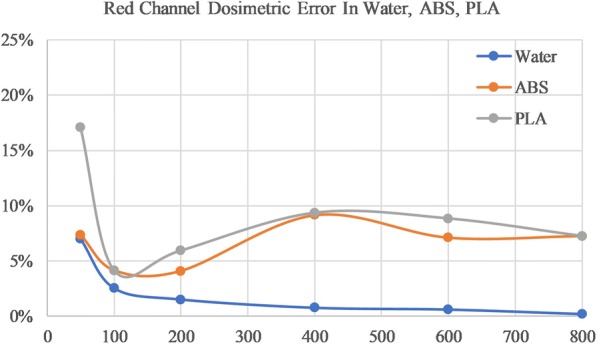
Error between measured and planning dose for red channel film in Water (blue), ABS (orange), and PLA (grey)

**Fig. 6:**
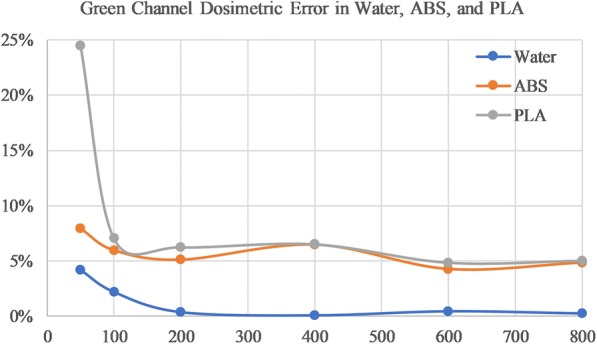
Error between measured and planning dose for green channel film in Water (blue), ABS (orange), and PLA (grey)

The red channel calibration curve from water (in Table 1) was used to fit NOD values for ABS and PLA film of increasing dose levels. Table 3 shows the measured dose and the percent error from the expected dose based on the red channel fit. Again, mean error was divided into low and high dose to account for inaccurate film response for doses at and below 100 cGy. A mean error of 6.9% and 7.8%, for ABS and PLA respectively for high doses using the red channel as seen in Table 3. The green channel measured a high dose mean error of 5.2% and 5.7% for ABS and PLA respectively. Table 4 displays green channel dose measurements. Figures 5 and 6 demonstrate how error changes with increasing dose for water, ABS, and PLA.

**Table 3:** Red Channel Dose Residual Error in Water, ABS and PLA

TPS Dose (cGy)	Water Error (%)	ABS Error (%)	PLA Error (%)
50	7.0	−7.4	−17.1
100	2.5	−4.1	−4.1
200	1.5	−4.1	−5.9
400	−0.8	−9.2	−9.4
600	−0.6	−7.1	−8.8
800	0.2	−7.3	−7.3
Low Dose Mean	4.8	5.8	10.6
High Dose Mean	0.8	6.9	7.8

The red channel calibration is used to estimate dose to water based on ABS and PLA NOD values. The error between TPS dose and measured dose is displayed for each dose level, as well as the combined absolute mean error for low dose (≤100 cGy) and high dose (> 100 cGy) ranges

**Table 4:** Green Channel Dose Residual Error in Water, ABS, and PLA

TPS Dose (cGy)	Water Error (%)	ABS Error (%)	PLA Error (%)
50	4.2	−7.9	−24.5
100	−2.2	−5.9	−7.0
200	−0.4	−5.1	−6.3
400	0.1	−6.5	−6.5
600	0.4	−4.3	− 4.9
800	−0.2	−4.8	−5.0
Low Dose Mean	3.2	6.9	15.8
High Dose Mean	0.3	5.2	5.7

The green channel curve is used to estimate dose to water based on ABS and PLA NOD values. The error between TPS dose and measured is displayed for each dose level, as well as the combined absolute mean error for low dose (≤100 cGy) and high dose (> 100 cGy) ranges

## Discussion

### Uncertainty analysis

Uncertainties reported in AAPM Task Group 43 were both statistical, random (type A) and systematic, nonrandom (type B) [13]. Also suggested by AAPM Task Group 43, the combined uncertainty was estimated using a simple root sum of squares of individual components. Our uncertainty analysis was broken into two parts; obtaining dose from NOD, and film exposure. Timer error and calibration curve fit are dose dependent errors, therefore both low and high dose uncertainties were estimated. Table 5 summarizes sources of uncertainty and combined uncertainty for high and lose dose measurements in film.

**Table 5:** Uncertainty Analysis

I. Uncertainty in Determining Dose from NOD				
	*Low Dose*		*High Dose*	
*Uncertainty Description*	*Type A (%)*	*Type B (%)*	*Type A (%)*	*Type B (%)*
Scanning Consistency	0.1		0.1	
Film uniformity		1.0		1.0
ROI Size		1.5		1.5
Calibration Curve Fit		4.8*		0.8*
Combined Uncertainty	0.1	5.1	0.1	2.0
Total Uncertainty	5.1		2.0	
II. Uncertainty in Film Exposure				
	*Low Dose*		*High Dose*	
*Uncertainty Description*	*Type A (%)*	*Type B (%)*	*Type A (%)*	*Type B (%)*
Repeatability	0.1		0.10	
Distance to Source		4.0		4.0
Lack of Scatter Equilibrium		5.0		5.0
Source Strength		1.5		1.5
Exposure Time		0.8*		0.1*
Combined Uncertainty	0.1	6.6	0.10	6.6
	6.6		6.6	
III. Total Uncertainty	8.4		6.9	

*Indicates differences between high and low dose uncertainty

Individual sources of uncertainty were determined for Dose from NOD Procedure (I) and consequential Film Exposure (II). Both random, statistical (Type A) and non-random, systematic (Type B) errors were determined. A root sum of squares is calculated to obtain combined, and total uncertainties

### Determining dose from NOD

Scanning consistency added minimal statistical uncertainty, less than 0.1%. Scanning consistency was determined from the difference between six separate scans of the same film piece. Film uniformity was determined by measuring difference between four quadrants of a background film sample. An additional source to consider was the ROI size. Decreasing the ROI size would reduce the amount of data sampled and may overestimate the optical density. This error was again more prominent at lower dose levels, with a low signal to noise ratio. Previous literature has found little uncertainty for a ROI 4 × 4 mm^2^ or larger [15, 16]. Our 9 × 9 pixel ROI was equivalent to a 4.1 × 4.1 mm^2^ ROI. Compared to a 2.3 × 2.3 mm^2^ ROI, net optical density for our larger ROI differed by about 1.5% for red channel water measurements.

With the obtained fitting parameters, the water calibration curves gave residual error for dose, especially at low dose levels as outlined in Table 2. For this reason, a systematic uncertainty error was added to the analysis for low and high dose levels. Combined uncertainty from Dose to OD procedure was estimated to be 5.1 and 2.0% for low and high dose, respectively.

### Film exposure

Positional accuracy between the film and source was a large cause of dosimetric error. In water measurements, a slight bend in the film may cause the source to be incorrectly placed up to 1 mm. Although a double catheter setup was used to minimize film position error, it was still present. Source film position error was determined by relating exposure at 5 cm to exposure at 5.1 cm away by the inverse square relationship. For example, a 1 mm uncertainty would lead to a 4% error, for water measurements at 5 cm.

Scatter equilibrium is an important consideration in superficial brachytherapy. At the skin surface there are less photons scattered from surrounding tissue, causing a smaller dose to the target. For ABS and PLA measurements, there was only 10 cm of solid water surrounding the 3D printed block/catheter (Fig. 4). Previous literature has suggested that full scatter equilibrium is achieved with at least 40 cm of water surrounding an Ir-192 source, otherwise the radial dose function can differ by 5–10%, thus under-dosing the target [17]. With the knowledge that our ABS and PLA film exposures did not achieve full scatter equilibrium, 5% systematic uncertainty is added. While this adds large uncertainty, it is a clinically relevant consideration. In practice full scatter equilibrium is not achievable for superficial HDR brachytherapy treatments within 5 mm of the skin surface [18]. Future studies should be completed to address loss of scatter conditions in superficial brachytherapy dosimetry.

Timer error added minimal uncertainty, but still was considered in our analysis. Low doses required smaller dwell times; thus, a higher proportion of timer error. For example, a 50 cGy dose to water required a dwell time of 50 s. A 0.4 s timer uncertainty would lead to a 0.8% timer error, while an 800 cGy dose and 800 s dwell time would produce an error of only 0.05%. Total uncertainty for low and high dose measurement in film was 8.4 and 6.9%, respectively.

### Clinical relevance of results

In HDR brachytherapy, most prescribed fractionation schemes require doses of 500 cGy per fraction, or more [2, 3]. Our results showed that at doses beyond 200 cGy, dosimetric error was near the expected level of uncertainty. The green channel proved to have the most sensitivity by measuring dose within 6% of the expected dose at high dose levels. This finding was in alignment with previous literature [4, 10]. The red channel showed less sensitivity but still within 8% at high dose levels for both PLA and ABS.

## Conclusions

A calibration curve was accurately obtained for water, and doses to water were compared to doses to 3D printed PLA and ABS plastics. For the calibration curve fit in water at low dose levels (100 cGy and below), there was an error up to 4.8% in the red channel. This error in the calibration curve fit is minimized for doses beyond 200 cGy. At high dose levels (200 cGy and higher), the calibration curve fit in water presented a mean error of 0.8, 0.3, and 0.7% in respective red, green, and blue channels. An accurate setup, with high positional accuracy is necessary to ensure that a quality calibration curve is acquired.

The results estimated the uncertainties and errors in measured doses when using 3D printed PLA and ABS plastics as summarized in Table 6. Distance and lack of scatter equilibrium were the largest source of uncertainty in our measurements. Measured doses in ABS and PLA were within the expected range of uncertainty.

**Table 6:** Summary of Dose Errors

Dose Level	Material	Color Channel	Error (%)
(%Uncertainty)			
High	Water	Red	0.8
(6.86%)		Green	0.3
	ABS	Red	−6.9
		Green	−5.2
	PLA	Red	−7.8
		Green	−5.7
Low	Water	Red	4.8
(8.36%)		Green	3.2
	ABS	Red	−5.8
		Green	−6.9
	PLA	Red	−10.6
		Green	−15.8

Table 6 Legend: Summary of error values for red and green channels in Water, ABS, and PLA at high and low dose levels with uncertainties

These results have shown that 3D printed PLA and ABS plastics can be used in lieu of water for film calibration for HDR Iridium-192 brachytherapy sources, however systematic uncertainties related to source position, and scatter equilibrium conditions due to the phantom size should be carefully accounted for.

## Abbreviations

AAPM: American association of physicists in medicine
ABS: Acrylic butadiene styrene
ADCL: Accredited dosimetric calibration laboratory
HDR: High dose rate
NOD: Net optical density
PLA: Polymethylmethacrylate
ROI: Region of interest
TPS: Treatment planning system

## References

[1] Jones EL, Baldion AT, Thomas C, Burrows T, Byrne N, Newton V, Aldridge S (2017). Introduction of novel 3D-printed superficial applicators for high-dose-rate skin brachytherapy. Brachy.

[2] Ouhib Z, Kasper M, Perez-Calatayud J, Rodriguez S, Bhatnagar A, Pai S, Strasswimmer J (2015). Aspects of dosimetry and clinical practice of skin brachytherapy: the American brachytherapy society working group report. Brachy.

[3] Guinot JL, Rembielak A, Perez-Calatayud J, Kovacs G (2018). GEC-ESTRO ACROP recommendations in skin brachytherapy. Rad Onc.

[4] Ayoobian N, Asl AS, Poorbaygi H, Javanshir MR (2015). Gafchromic film dosimetry of a new HDR Ir-192 brachytherapy source. J Appl Clin Med Phys.

[5] Mathot M, Sobczak S, Hoornaert MT (2014). Gafchromic film dosimetry: four years experience using FilmQA pro software and Epson flatbed scanners. Physca Medica.

[6] Craft DF, Kry SF, Balter P, Salehpour M, Woodward W, Howell RM. Material matters: analysis of density uncertainty in 3D printing and its consequences for radiation oncology. Med Phys. 2018. 10.1002/mp.12839. 10.1002/mp.12839 29493803

[7] León-Marroquín EY, Herrera-González JA, Camacho-López MA, Villarreal-Barajas JE, García-Garduño OA. Evaluation of the uncertainty in a EBT3 film dosimetry system utilizing net optical density. J Appl Clin Med Physv17. 2016:466–81. 10.1120/jacmp.v17i5.6262 PMC5874103 27685125

[8] Massillon JL, Chiu-Tsao ST, Domingo-Munoz I, Chan MF (2012). Energy dependence of new Gafchromic EBT3 film: dose response curves for 50 kV, 6 and 15 MV X-ray beams. IJMPCERO.

[9] Reinhardt S, Hillbrand M, Wilkens JJ, Assmann W (2012). Comparison of Gafchromic EBT2 and EBT3 films for clinical photon and proton beams. Med Phys.

[10] Casanova Borca V, Pasquino M, Russo G, Grosso, Cante D, Sciacero P, Girelli G, La Porta MR, Tofani S (2013) GAFCHROMIC EBT3 film for IMRT dose verification. J Appl Clin Med Phys. 10.1120/jacmp.v14i2.4111 PMC5714357 23470940

[11] Niroomand-Rad A, Blackwell CR, Coursey BM, Gall KP, Galvin JM, McLaughlin WL, Meigooni AS, Nath R, Rodgers JE, Soares CG (1998). Radiochromic film dosimetry: recommendations of AAPM radiation therapy committee task group 55. Med Phys.

[12] Nath R, Anderson LL, Luxton G, Weaver KA, Williamson JF, Meigooni AS (1995). Dosimetry of interstitial brachytherapy sources: recommendations of the AAPM radiation therapy committee task group no. 43. Med Phys.

[13] Rivard MJ, Coursey BM, DeWerd LA, Hanson WF, Huq MS, Ibbott GS, Mitch MG, Nath R, Williamson JF (2004). Update of AAPM task group no. 43 report: a revised AAPM protocol for brachytherapy dose calculations. Med Phys.

[14] Micke A, Lewis DF, Yu X (2011). Multichannel film dosimetry with nonuniformity correction. Med Phys.

[15] Bouchard H, Lacroix F, Beaudoin G, Kawrakow I (2009). On the characterization and uncertainty analysis of Radiochromic film dosimetry. Med Phys.

[16] Sendani NG, Karimian A, Ferreira C, Alaei P (2018). Technical note: impact of region of interest size and location in Gafchromic film dosimetry. Med Phys.

[17] Perez- Calatayud J, Granero D, Ballester F. Phantom size in brachytherapy source dosimetric studies. Med Phys. 2004;31(7). 10.1118/1.1759826 15305460

[18] Melhus CS, Rivard MJ (2006). Approaches to calculating AAPM TG-43 brachytherapy dosimetry parameters for 137Cs, 125I, 192Ir, 103Pd, and 169Yb sources. Med Phys.

